# Impaired flow-mediated dilation response and carotid intima-media thickness in patients with type 1 diabetes mellitus with a mean disease duration of 4.1 years

**DOI:** 10.1590/2359-3997000000281

**Published:** 2017-06-23

**Authors:** Lúcia Helena Bonalume Tacito, Antonio Carlos Pires, Juan Carlos Yugar-Toledo

**Affiliations:** 1 Departamento de Medicina Faculdade de Medicina de São José do Rio Preto São Paulo SP Brasil Disciplina de Endocrinologia, Departamento de Medicina, Faculdade de Medicina de São José do Rio Preto (Famerp), São Paulo, SP, Brasil; 2 Instituto de Cardiologia e Endocrinologia de São José do Rio Preto São Paulo SP Brasil Endocor – Instituto de Cardiologia e Endocrinologia de São José do Rio Preto, São Paulo, SP, Brasil

**Keywords:** Nitric oxide, carotid intima-media thickness, diabetes mellitus, type 1, cardiovascular diseases, diabetes complications

## Abstract

**Objective:**

This study aimed at assessing the endothelial function in patients with Type 1 diabetes (T1DM) using flow-mediated dilation (FMD) response and carotid artery intima-media thickness (CIMT).

**Materials and methods:**

This study enrolled 32 T1DM patients (mean disease duration 4.1 years) and 28 age-matched controls (CTL Group). Endothelial function and CIMT were assessed with high-resolution ultrasound using standardized offline measurements.

**Results:**

FMD was significantly lower in patients in the T1DM Group (8.9 ± 3.2%) compared with those in the CTL Group (13.3 ± 4.3%; P-value < 0.0001). Similarly, CIMT differed significantly between T1DM patients (0.525 ± 0.03 mm) and controls (0.508 ± 0.03 mm; P-value = 0.041). Even though, the values are within the normal range for age.

**Conclusions:**

Patients with T1DM have impaired endothelial function characterized by reduced FMD when compared to controls. However, vascular remodeling as seen by increases in CIMT was not found in this study.

## INTRODUCTION

Cardiovascular disease (CVD) is the leading cause of morbidity and mortality in patients with diabetes. Macro- and micro-vascular complications are involved in the pathophysiology of CVD and the increased risk of developing atherosclerosis in this population (1,2). Type 1 diabetics have a six- to ten-times higher risk of premature cardiovascular death (before the age of 60 years) than nondiabetic individuals (3). Studies have also shown that the mortality rate due to cerebrovascular disease is higher in all age groups of type 1 diabetics (3). However, the traditional risk factors for coronary heart disease do not entirely explain this higher risk. A probable association between type 1 diabetes mellitus (Type 1 DM) and CVD has been attributed to chronic uncontrolled hyperglycemia, inflammation, endothelial dysfunction (ED), and subclinical manifestations of vascular disease (4,5).

ED and increases in the common carotid intima-media thickness (CIMT) are early markers of atherosclerosis in individuals with risk factors for CVD (6). These markers have played a central role in the pathophysiology of macro-vascular complications in Type 1 DM. Incipient atherosclerosis and ED can be investigated noninvasively in different vascular beds using mechanical or pharmacological stimulation (7).

Flow-mediated dilation (FMD) can be induced by reactive hyperemia after compression and decompression of the brachial artery. The increased flow resulting from this maneuver causes an increase in the “shear stress” on the vascular wall, which is detected by endothelial mechanical sensors. In normal arteries, this leads to the production and release of vasodilatory substances, such as nitric oxide (NO), by the endothelium. Thus, the increase of NO bioavailability promotes NO-mediated artery dilation, that is, endothelium-dependent dilation (8). A decrease in endothelium-dependent dilation is interpreted as a functional change of endothelial cells, mediated via the nitric oxide – cyclic guanosine monophosphate (NO – cGMP) pathway. In contrast, NO donors, such as nitroglycerin, acting directly on the vascular smooth muscle, promote endothelium-independent dilation.

One important tool in clinical research is high-resolution ultrasound, which allows the determination of percentage variations of the diameter of the brachial artery. The results of this test are a measure of the NO-mediated (endothelium-dependent) dilation. This technique involves reactive hyperemia and shear stress to stimulate FMD (9).

The endothelial vasodilator mechanism is mediated by vasoactive substances, mainly by NO (a physiological antagonist of endogenous vasoconstricting substances such as catecholamines, angiotensin II and endothelin-1), but also by prostacyclin, bradykinin, endothelium-derived hyperpolarizing factor (EDHF), monooxygenase metabolites, and others (10-12).

Endothelial cells release NO not only into the layer of smooth muscle cells, but also into the lumen of the blood vessel. This vasodilator is formed from the terminal nitrogen of the guanidine group of the L-arginine amino acid by the action of the endothelial NO synthase enzyme (eNOS) which is constitutive (NO synthase III); this is dependent on the intracellular concentration of calcium ions and calmodulin and requires reduced levels of nicotinamide-adenine-dinucleotide phosphate (NADPH) and tetrahydrobiopterin (4HB) for optimal activity (13). NO also inhibits platelet and leukocyte adhesion to the endothelium, has an inhibitory action on platelet aggregation (synergistically with prostacyclin), inhibits the proliferation of smooth muscle cells and modulates the production of adhesion molecules, and endothelin-1 that are involved in the pathophysiology of atherosclerosis (14).

The measurement of the CIMT in the anterior and posterior walls using high-resolution ultrasound is another commonly used research tool employed to detect vascular remodeling at an early stage; this is believed to be the first structural change in atherosclerosis. Since the initial works by Pignoli and cols. in 1986 (15), several population and case-control studies (16,17) have demonstrated the excellent safety, reliability, reproducibility and applicability of this technique in studies on primary prevention and cardiovascular risk stratification. Today, this method is recognized as a marker of risk for myocardial infarction, stroke, and peripheral artery disease (18). This study aims to assess endothelial function using FMD and measurements of the CIMT in patients with Type 1 DM and correlate the findings with metabolic parameters.

## MATERIALS AND METHODS

This case-control study enrolled 60 individuals. Thirty-two were diabetics (Type 1 DM – 20 female and 12 male) treated in the Diabetes Outpatient Clinic of the Medicine School in São José do Rio Preto (Famerp) with a mean time after diagnosis of 4.1 years, and 28 were apparently healthy volunteer controls (CTL Group – 20 female and 8 male).

Individuals with primary and secondary forms of hypertension, impaired renal function, or dyslipidemia, smokers and those with any other major disease were excluded.

This research project was approved by the Research Ethics Committee of the Medicine School in São José do Rio Preto (Famerp). All patients were followed clinically by experts and received treatment for their disorders according to routine clinical standards and norms. The nature of the study was carefully explained to patients and all individuals, after agreeing to participate in the study, signed informed consent forms. All patients and volunteers who accepted to participate filled out a standard questionnaire. The study was conducted in compliance with the principles of the Declaration of Helsinki.

### Flow-mediated dilation

High-resolution ultrasound was used to evaluate the endothelium-dependent function of a medium-caliber artery (brachial artery) after applying the compression/decompression test (occlusion for five minutes). This method has been validated and is standardized according to the International Brachial Artery Reactivity Task Force Guidelines for the Ultrasound Assessment of Endothelial-dependent Vasodilation of the Brachial Artery (9). A Philips HDI ultrasound equipment with a high-resolution 5-12 MHz vascular linear transducer was used connected to a microcomputer to study the vascular function dynamically. All scanned images were stored on a compact disc for future analysis by two independent observers. The variability between the arterial diameter measurements should be less than 2%, and intra-observer differences less than 1%, as was seen in this study.

The brachial artery diameter was measured using offline image analysis software (M’ATh – Metris France). Measurements were taken between the arterial lumen-wall interface of the front and posterior walls at the end of diastole. The mean diameter was calculated in four cardiac cycles identified using the R wave of the electrocardiogram (ECG). Percentage changes in the brachial artery diameter were calculated compared to the first baseline diameter (100%) according to the formula: FMD = [(diameter after decompression – baseline diameter) / baseline diameter] x 100.

### Common carotid intima-media thickness measurements

Measurements of the CIMT of the anterior and posterior walls were achieved using high-resolution ultrasound. This method is established and standardized by the report of the 34^th^ Bethesda Conference Task Force #3 Noninvasive Measurement of Atherosclerosis (19). The brightness of the examination room was controlled, and the room temperature was set at 24°C.

In order to perform the examination, the patient was positioned in the supine position with the head flexed slightly toward the side opposite to that being examined. An image of the vessel was positioned on the screen so that the cephalic portion was on the left. Care was taken not to excessively compress the tissue with the transducer so that the venous structures anterior to the carotid artery were not collapsed. The lumen-intima interface and the adventitia-media interface were clearly defined with the proper use of gain control, correction of angle and slope of the sampling box, and its amplification.

A Philips HDI ultrasound equipment with a high-resolution 5-12 MHz vascular linear transducer connected to a microcomputer was used to measure the CIMT automatically. The image acquisition protocol for the distal segment of the common carotid artery was standardized to a minimum of 100 measurement points or a longitudinal length of at least 1.0 cm of the artery excluding the carotid bulb (20).

The CIMT was measured in the distal segment during four cardiac cycles identified by the R wave of the ECG using image analysis software (M’ATh-Metris – France) which allows measurement of the CIMT from stored images. Analysis is based on the gray-scale density and a specific tissue recognition algorithm, which allows automatic measurement without depending on the observer. The variability between the CIMT measurements should be less than 2%, as was seen in this study.

### Statistical analysis

All calculations were performed using SPSS for Windows, version 17.0 (SPSS Inc., Chicago, IL, USA) and the Graph Pad Prism 5 Statistical package (CA) was used for all analyses. The results of the continuous variables with normal distribution are presented as means and standard deviations; comparative analysis employed the unpaired t-test. Variables with non-Gaussian distributions are presented as medians with the Mann-Whitney test being used for comparative analysis. Multivariate logistic regression was performed to determine predictors of ED and increases in the CIMT. *P*-value < 0.05 was considered significant.

## RESULTS

The main demographic, clinical and anthropometrical characteristics of the study groups are presented in Table 1. The mean age in the control group was higher than in patients with DM1. However, no statistically significant difference was found between the median ages of both groups. There were no statistical differences between the Type 1 DM and CTL Groups in regards to body mass index (BMI), total cholesterol, triglycerides, and high-density lipoprotein (HDL) cholesterol levels. The low-density lipoprotein cholesterol (LDL) and TSH plasma levels were increased in Type 1 DM when compared with the CTL Group (*P*-value = 0.0005 and *P*-value = 0.035, respectively). However, despite the difference in mean values of LDL cholesterol, no correlation was found between the LDL cholesterol and FMD in the control and DM1 groups. Microalbuminuria levels were increased in Type 1 DM when compared with the CTL Group (*P*-value < 0.0001). Nonetheless, even with the difference in mean values of albuminuria, no correlation was found between the microalbuminuria and FMD in the control and DM1 groups. However, as expected, there was a correlation between elevated levels of glycemia and microalbuminuria in patients with DM1 (R = 0.48; *P*-value = 0.038).

**Table 1:** Demographic, clinical and anthropometrical characteristics of the Type 1 diabetes mellitus (Type 1 DM) and Control Groups

Parameters	Type 1 DM	Controls	*p*-value
Age	17.25 ± 4.43	20.11 ± 5.62	0.03*
Gender F/M	20/12	20/8	NS
Body mass index	21.71 ± 3.14	21. 34 ± 2.32	NS
Duration of Type 1 DM	4.14 ± 1.97	-	-
Glycemia	170.1 ± 77.99	79.82 ± 11.89	< 0.0001^†^
HbA1c	9.95 ± 2.97	5.47 ± 0.43	< 0.0001^†^
Total cholesterol	160.7 ± 32.13	145.7 ± 19.91	0.057
Triglycerides	68.12 ± 37.24	67.75 ± 19.91	0.96
HDL cholesterol	57.19 ± 16.75	65.81 ± 13.44	0.056
LDL cholesterol	92.15 ± 28.19	65.75 ± 17.67	0.0005^#^
VLDL cholesterol	13.81 ± 7.38	13.75 ± 4.27	0.97
Microalbuminuria	48.40 ± 9.3	13.90 ± 1.8	< 0.0001^†^
TSH	3.48 ± 1.63	2.65 ± 1.11	0.035^‡^
FT4	1.17 ± 0.18	1.11 ± 0.21	0.27

* P-value < 0.03 (absolute difference between Type 1 diabetes mellitus and Control groups).

^†^ P-value < 0.0001 (absolute differences between Type 1 diabetes mellitus and control groups).

^#^ P-value = 0.0005 (absolute difference between Type 1 diabetes mellitus and control groups).

^‡^ P-value = 0.035 (absolute difference between Type 1 diabetes mellitus and control groups).

The absolute variations in the diameter of the brachial artery and the relative difference between Type 1 DM and CTL groups during the assessment of endothelium-dependent vascular function and CIMT using high-resolution ultrasound are presented in Table 2.

**Table 2:** The absolute and relative differences in the diameter of the brachial artery and the carotid intima-media thickness comparing Type 1 diabetes mellitus (Type 1 DM) and Control Groups

	Type 1 DM			Controls		
	
	Mean	SD	95% CI	Mean	SD	95% CI
Brachial artery diameter (mm) – BL	3.23	0.36	2.70 – 3.93	3.13	0,37	2.58 – 3.97
Brachial artery diameter (mm) – FMD	3.52	0.42	2.91 – 4.29	3.54	0.406	2.96 – 4.30
Absolute variation in the diameter of the brachial artery [FMD-BL] (mm)	0.29*	0.11	0.15 – 0.52	0.41	0.13	0.20 – 0.62
Relative difference (%)	8.94^#^	3.21	4.62 – 14.65	13.27	4.22	7.10 – 20.90
Carotid IMT (mm)	0.525^‡^	0.03	0.467 – 0.599	0.508	0.03	0.456 – 0.566

SD: standard deviation; CI: confidence interval; mm: millimeters; BL: baseline; FMD: flow-mediated-dilation; Relative differences %: percentage from the formula ([FMD-BL/BL]*100); IMT: intima-media thickness.

* P-value = 0.003 (absolute difference in the diameter of the brachial artery between Type 1 diabetes mellitus and Control groups).

^#^ P-value < 0.0001 (relative difference in the diameter of the brachial artery between Type 1 diabetes mellitus and Control groups).

^‡^ P-value: 0.41 (absolute difference in the common carotid artery intima-media thickness – IMT – between Type 1 diabetes mellitus and Control groups).

**Figure 1:**
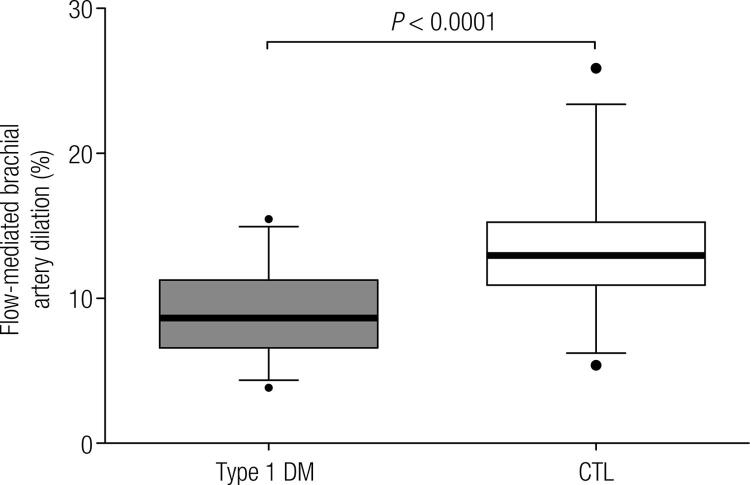
Flow-mediated dilation (FMD) of the brachial artery in Type 1 diabetes mellitus (Type 1 DM) patients and Control (CTL) groups. The Type 1 DM group had a significant reduction in FMD (P-value < 0.0001).

The results of the assessment of endothelium-dependent vascular function using high-resolution ultrasound and mechanical stimulation (compression-decompression) of the brachial artery (FMD) are presented in Figure 1. The values found for the FMD in the Type 1 DM and CTL Groups were 8.94 ± 3.21% and 13.27 ± 4.22%, respectively (*P*-value < 0.0001).

**Figure 2:**
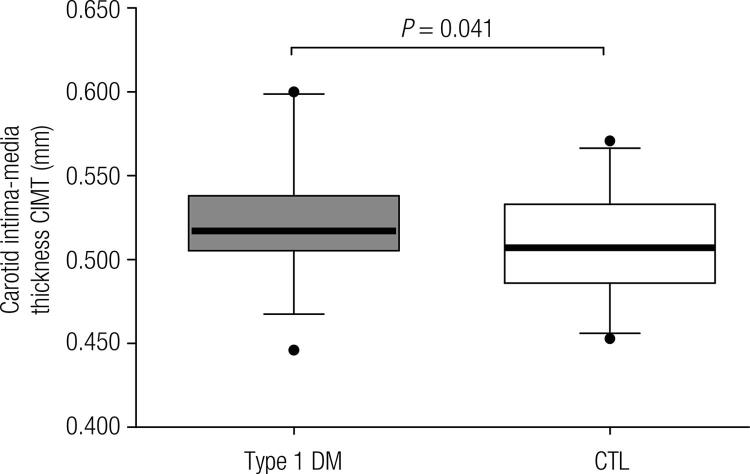
Common carotid artery intima-media thickness (CIMT) in Type 1 diabetes mellitus (Type 1 DM) patients and Control (CTL) groups. The Type 1 DM group had a significantly greater CIMT (P-value = 0.041). Even though, the values are within the normal range for age.

The results of the measurement of the CIMT are presented in Figure 2. The mean CIMT in the Type 1 DM and CTL Groups were 0.525 ± 0.03 mm and 0.508 ± 0.03 mm, respectively with statistical significance (*P*-value = 0.041). Even though, the values are within the normal range for age.

## DISCUSSION

The main results of this study show: 1) a reduction in the FMD in Type 1 DM patients in relation to controls individuals and 2) the absence of the vascular remodeling as seen by the increased CIMT for age.

This study of a population of young Brazilian individuals with Type 1 DM demonstrates that impaired vascular function (reduced FMD of the brachial artery) is a manifestation of vascular involvement early in the evolution of Type 1 DM preceding vascular remodeling characterized by increased CIMT, an early marker of subclinical atherosclerosis. The duration of diabetes in this population was less than five years (mean disease duration 4.1 years). Wiltshire and cols. (21), who evaluated 36 Type 1 DM patients with a mean age of 16 years by comparing them to 20 healthy control individuals found reduced FMD only in patients with Type 1 DM. Similarly, on evaluating young adolescents with Type 1 DM, Singh and cols. (22) demonstrated that changes in the endothelial function occur within the first decade after the onset of Type 1 DM. However, morphological alterations of the CIMT appear later due to, according to the authors, prolonged and chronic exposure to hyperglycemia and the metabolic changes related to this clinical condition. Hurks and cols. (23) investigated the endothelial function of Type 1 DM patients and controls, matched by age and gender (aged 16-36 years), and without subclinical atherosclerosis or risk factors for CVD. Duration of diabetes was 9.2 ± 5.3 years. The authors demonstrated that even diabetics with moderate metabolic control [glycated hemoglobin (HbA1c): 7.6 ± 1.0] have changes in FMD, and they concluded that even without preclinical atherosclerosis, the endothelial function is already affected as can be confirmed by assessing the endothelial function using high-resolution ultrasound.

The pathophysiology of ED and of macro- and micro-vascular changes in Type 1 DM involve multiple factors such as age at onset, time of evolution of the disease, and the presence of risk factors for heart disease such as smoking, hypertension and dyslipidemia. Thus, good metabolic control, which is difficult to achieve, seems to be insufficient to prevent the development of micro- and macro-vascular complications (24).

Hyperglycemia leads to increased oxidative stress and the formation of non-enzymatic glycation end products. These, in turn, increase the inactivation of NO and promote the oxidative modification of lipoproteins (25). Moreover, the metabolism of lipoproteins is altered in Type 1 DM leading to hypertriglyceridemia associated with decreased concentrations of HDL cholesterol and increases in small and dense particles of LDL cholesterol with normal or slightly increased total cholesterol. This constitutes the so-called atherogenic profile; this condition, associated with ED, appears to increase the susceptibility of young people with Type 1 DM to the harmful effects of LDL cholesterol and the occurrence of early-onset atherosclerosis (26). On the other hand, through the elevation of intracellular calcium, hyperglycemia stimulates the synthesis of NO which, in the presence of peroxide anions, is quickly converted to peroxynitrite, a potent oxidizing molecule, thus contributing to the perpetuation of oxidative stress (27).

Some studies, such as the one by Sibal and cols. (28), showed impairment of endothelial function and an increase in CIMT in young people with Type 1 DM without macro-vascular disease or microalbuminuria. However, their study included 62% of individuals with retinopathy, 24% of smokers and metabolic control outside the goals recommended by guidelines (mean HbA1c = 8.5%). Previously, Larsen and cols. (29) reported that an increased CIMT in Type 1 DM is significantly associated with elevated levels of HbA1c (r^2^ = 0.77; *P*-value < 0.0001 adjusted for age) in women with Type 1 DM while no correlation was observed in men.

The atherogenicity in Type 1 DM has been widely recognized. However, there is controversy over the timing of the early markers of atherosclerosis, such as vascular remodeling as seen by an increased CIMT, in young people; this change is not present in the first years of the development of this metabolic disorder. However, the results of the Epidemiology of Diabetes Interventions and Complications (EDIC) Study (30) show that intensive insulin therapy delays increases in CIMT more than conventional therapy. Nevertheless, many questions remain unanswered such as the minimum time of exposure required for a clinical event to happen, the specific determinants in Type 1 DM for vascular damage, and whether the atherosclerotic process is already active in the prepubertal period.

Studies on the CIMT in children and adolescents have conflicting results; some studies show an increase in the CIMT in diabetic patients, while others do not (31-33). The seemingly contradictory results may depend on the different ultrasound techniques used as well as on the different populations studied. However, the use of automated programs that measure the CIMT reduces the variability related to human error and allows comparisons between studies. Our results on the CIMT measured using an automated system comparing Type 1 DM and controls are similar to other publications (22,33).

However, recent publications such as the study by Bradley and cols. (34) that included a sample of patients with longer disease duration (on average 6.2 years) demonstrated a higher blood pressure, impaired endothelial function and increased arterial stiffness, altered myocardial velocities and strain. Similar results were observed by Abd Al Dayem and cols. (35) who included patients with longer duration of type 1 DM (9.4 ± 2.9 years). These authors demonstrated that the mean CIMT was significantly higher, whereas the FMD and FMD – nitrate mediated dilatation (NMD) ratio was significantly lower in diabetics; CIMT had a significant negative correlation with the FMD and FMD – NMD ratio. CIMT had a significant positive correlation with left ventricular end diastolic dimension, inter-ventricular septum thickness, peak mitral flow velocity during early diastole/peak mitral flow velocity during late diastole, left ventricular mass, and left ventricular mass index. In addition, CIMT had a significant correlation with waist circumference, waist/height ratio, albumin/creatinine ratio, total cholesterol, and triglyceride. Obviously, it is expected that with this period of exposure to type 1 DM there will be increased cardiovascular and renal damage; we consider that the findings are relevant for this population.

Moreover, the work of Murat Ciftel and cols. (36), who studied 40 DM1 with disease duration < 5 years and 42 controls from Turkey, demonstrated, that the aortic strain (8.40 ± 2.98 vs. 20.12 ± 5.04; p-value < 0.001), aortic distensibility (7.36 ± 2.92 vs. 16.59 ± 4.25; p-value < 0.001) and FMD% (7.70 ± 2.83 vs. 11.33 ± 2.85; p-value < 0.001) were decreased, and CIMT (0.52 ± 0.09 mm vs. 0.47 ± 0.08 mm; p-value < 0.05) was increased in the diabetic group. Additionally, left ventricular lateral segment, right ventricular free-wall isovolumic relaxation time (IVRT) and myocardial performance index were found increased. Correlation analyses demonstrated a negative correlation between FMD and IVRT and MPI. These results corroborate our findings.

Ce and cols. (37) observed that ED in Type 1 DM is an early phenomenon that is relatively common in adolescents with recent onset of diabetes regardless of age, smoking, hypertension or hyperlipidemia. The reduction of flow-mediated vasodilation is particularly influenced by glycemic control and duration of disease. The authors suggest that medium-term and non-short-term glycemic control has a great influence on ED in the early years of Type 1 DM. Thus, we believe that, HbA1c at the time of the analysis of endothelial function was similar between those with and without ED (8.2 ± 0.9 vs. 8.0 ± 1.4%, respectively; P-value = 0.66), whereas the mean second-year HbA1c was significantly higher in individuals with ED compared to those without ED (9.6 ± 2.4 vs. 8.1 ± 1.3%, respectively; P-value = 0.048). Moreover, FMD was inversely correlated with mean second-year HbA1c (r = −0.287; P-value = 0.031) but not with mean first-year HbA1c (r = −0.126; P-value = 0.37).

In patients with less than 5 years of Type 1 DM, ED was a common finding (35.7%), but it was more prevalent in patients with longer duration of Type 1 DM (60%; P-value < 0.01).

The mechanism by which chronic hyperglycemia is associated with ED is complex and not fully understood. Oxidative stress, activation of the polyol pathway, activation of the protein kinase C system and the presence of advanced glycation end products are all potential mechanisms involved. The concept of metabolic memory was recently proposed by Ceriello and cols. (38), who pointed out that the mechanisms that propagate this phenomenon seem to be related to the non-enzymatic glycation process and the excess of reactive species of oxygen and nitrogen, originating at the glycated mitochondrial protein level and acting synergistically to maintain glucose-independent stress signaling.

However, a few points need to be stressed. This study of a Brazilian population of young individuals with Type 1 DM demonstrates that impaired functional vascular (reduced FMD of the brachial artery) is an early functional manifestation in the evolution of Type 1 DM and precedes vascular remodeling characterized by increased CIMT. These individuals had duration of diabetes of less than five years (mean disease duration 4.1 years). Even so, the necessity of new markers to monitor the integrity of cardiovascular health is undeniable because of the clinical importance of this pathology. A better understanding of this disease may help to create appropriate therapeutic strategies that limit the development of micro- and macro-vascular lesions and cardiovascular events.

Moreover, early detection of ED with prompt intervention may improve the treatment of patients with Type 1 DM at high cardiovascular risk. In fact, ED, characterized by an imbalance between vasodilator substances (particularly NO) and vasoconstrictor substances is an early process involved in the pathophysiology of several CVDs and is observed in humans with vascular risk factors. Furthermore, the presence of ED is predictive of future cardiovascular events in patients with vascular disease including preclinical Type 1 DM (39).

The significant difference found in our study in the CIMT between Type 1 DM and control subjects, even though the values were within the normal range for age, may represent an early stage of the atherosclerotic process in this group of patients. Identifying this condition may allow adaptations to treatment to improve glycemic control and combat cardiovascular risk factors thereby preventing future cardiovascular events (40).

In conclusion, endothelial dysfunction, characterized by reduced FMD, is an early marker of vascular involvement that appears within the first few years after the onset of Type 1 DM. However, increases in the CIMT, a preclinical marker of atherosclerosis that initiates the pathologic vascular remodeling process, is not present as early in the evolution of T1DM.
